# Exploring the mechanism of action of Shuangyang houbitong granules in the treatment of acute pharyngitis based on network pharmacology and molecular docking

**DOI:** 10.1097/MD.0000000000037674

**Published:** 2024-03-29

**Authors:** Jiying Zhou, Chuanqi Qiao, Yifei Gao, Haojia Wang, Jiaqi Li, Siyun Yang, Keyan Chai, Tong Zhao, Jiarui Wu

**Affiliations:** a Department of Clinical Chinese Pharmacy, School of Chinese Materia Medica, Beijing University of Chinese Medicine, Beijing, China.

**Keywords:** acute pharyngitis, molecular docking, network pharmacology, Shuangyang houbitong granules

## Abstract

**Background:**

Acute pharyngitis (AP) refers to the acute inflammation of the pharynx, characterized by swelling and pain in the throat. Shuangyang houbitong granules (SHG), a traditional Chinese medicine compound, have been found to be effective in providing relief from symptoms associated with AP.

**Methods:**

The chemical components of SHG were screened using Traditional Chinese Medicine Systems Pharmacology database, HERB database, and China National Knowledge Infrastructure. The targets of the granules were predicted using SwissTargetPrediction database. A network was constructed based on the targets of AP obtained from Genecards database, and protein–protein interaction analysis was performed on the intersection targets using STRING database. Key targets were screened for Gene Ontology and Kyoto Encyclopedia of Genes and Genomes enrichment analysis, and the binding activity of components and targets was predicted using AutoDockTools-1.5.7.

**Results:**

A total of 65 components of SHG that met the screening criteria were retrieved, resulting in 867 corresponding targets. Additionally, 1086 AP target genes were retrieved, and 272 gene targets were obtained from the intersection as potential targets for SHG in the treatment of AP. Molecular docking results showed that the core components genkwanin, acacetin, apigenin, quercetin can stably bind to the core targets glyceraldehyde 3-phosphate dehydrogenase, interleukin 6, tumor necrosis factor, serine/threonine protein kinase, tumor protein 53, and epidermal growth factor receptor.

**Conclusion:**

The research results preliminarily predict and verify the mechanism of action of SHG in the treatment of AP, providing insights for further in-depth research.

## 1. Introduction

Acute pharyngitis (AP) is characterized by the inflammation of the pharyngeal mucosa and submucosal tissue,^[1]^ leading to clinical symptoms such as redness, swelling, and pain in the pharynx. Additionally, patients might experience a foreign body sensation and pain while swallowing, significantly impacting daily life. Western medicine primarily attributes this condition to viral, bacterial, or fungal infections, with hemolytic streptococci and Staphylococcus aureus as the most common culprits.^[2,3]^ Treatment often involves antibiotics, which, despite their effectiveness, can result in noticeable side effects. Traditional Chinese medicine classifies AP as acute laryngitis and ascribes its etiology to lung and kidney *yin* deficiency, as well as excessive heat and toxins.^[4,5]^ Accordingly, remedies that nourish the kidney and lungs, provide heat and toxin relief, and soothe the throat are recommended.

**Figure 1.**
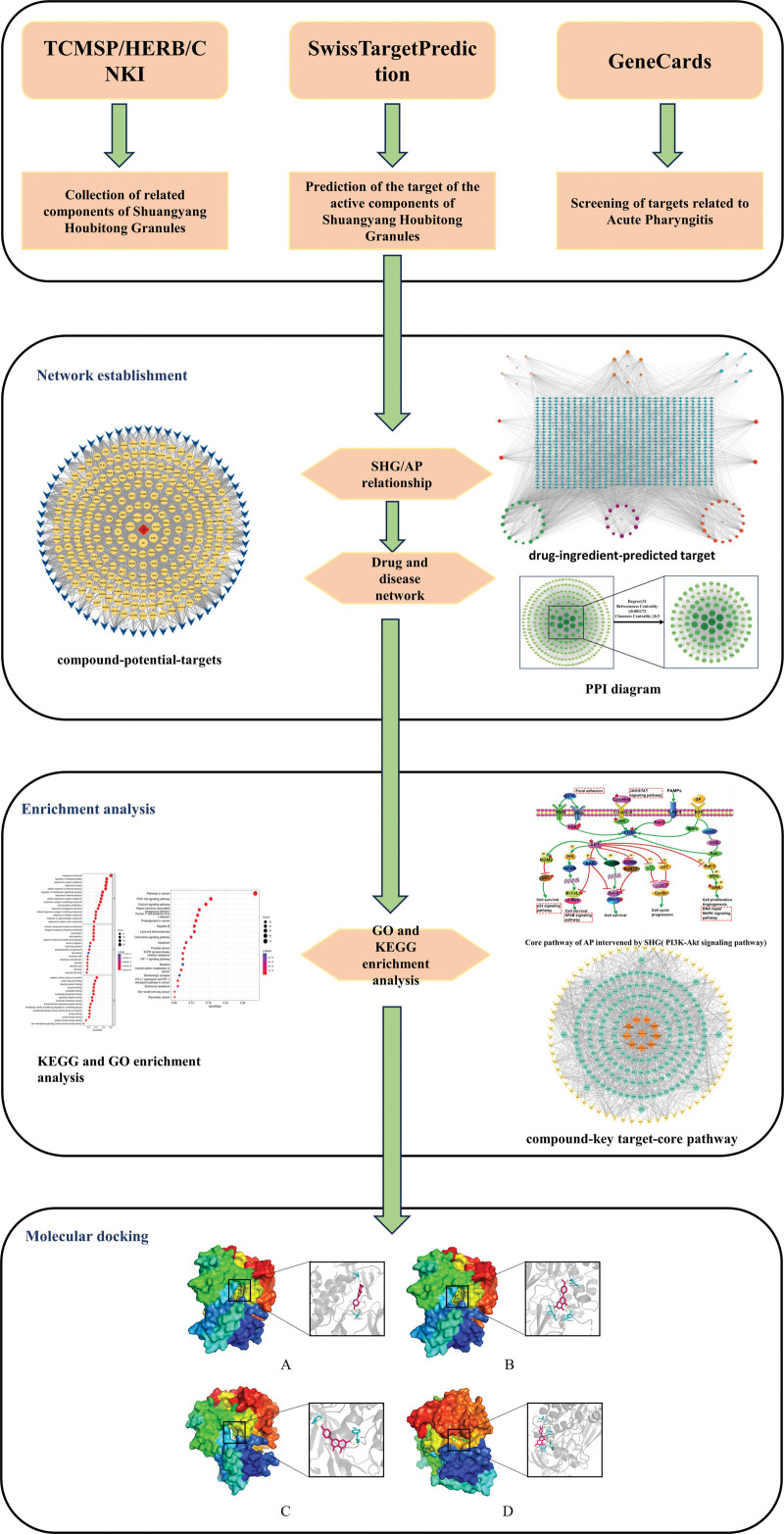
Network pharmacology research flow chart of SHG in the treatment of AP. AP = acute pharyngitis, SHG = Shuangyang houbitong granules.

Shuangyang houbitong granules (SHG), a traditional prescription from the Miao ethnic group, utilizes authentic medicinal materials from Guizhou such as *yangnainaiye, yeyanye*, and *Inula cappa*. These components exhibit the ability to remove wind toxins and alleviate sore throat. Furthermore, the granules display antibacterial and antiviral properties, exerting a specific inhibitory effect on Staphylococcus aureus, Group A Streptococcus, and Group B Streptococcus. The granules have been demonstrated to effectively inhibit viral growth and prevent influenza virus infection. They also possess anti-inflammatory, antipyretic, and antitussive effects. Clinical studies have shown that SHG significantly improves clinical symptoms and effectively treats AP.^[6,7]^ This study employs network pharmacology and molecular docking techniques to explore the mechanism of action of SHG for AP treatment, aiming to provide a foundation for further experiments and clinical applications. The flow chart of the network pharmacology method used in this study is shown in Figure 1.

## 2. Material and methods

### 2.1. Collection of related components of SHG

The Traditional Chinese Medicine Systems Pharmacology database and analysis platform (TCMSP)^[8]^ was used to search for the chemical composition of 7 traditional Chinese medicines, including *yeyanye, Inula cappa, Ardisia japonica, yangnainaiye, Bombyx Batryticatus, Schizonepetae Herba*, and *Mentha canadensis*. The selection criteria for the chemical components were an oral bioavailability of ≥30% and a drug-likeness of ≥0.18.^[9,10]^ Furthermore, additional databases such as China National Knowledge Infrastructure and Herb were searched to supplement the chemical components. The supplementary components were chosen based on high gastrointestinal absorption (GI absorption) in the Swiss ADME database and meeting two or more of the predicted drug-like properties (including Lipinski, Ghose, Veber, Egan, Muegge) as pharmacokinetic standards.^[11]^ This screening process facilitated identifying the chemical composition of SHG.

### 2.2. Prediction of the target of the active components of SHG

To determine the target genes of different chemical compositions, we utilized Pubchem database^[12]^ to collect the Smiles numbers of chemical compositions. Subsequently, these Smiles numbers were input into the SwissTargetPrediction^[13]^ database for target gene prediction. Finally, all targets with a probability greater than 0 were screened out.

### 2.3. Screening of targets related to AP

To screen the disease target genes of AP, the GeneCards database was searched using the keyword “Acute pharyngitis.” In this database, target genes with higher GIFtS (GeneCards Inferred Functionality Scores) values are considered more closely related to the disease. Based on previous experience, if there are multiple targets, they will be arranged in descending order of GIFtS values, and the targets with GIFtS scores greater than the median will be selected as the disease targets for AP.^[14]^ Subsequently, the targets of SHG were intersected with the disease targets, identifying the potential targets for treating AP with SHG.

### 2.4. Construction of “drug-compound-predicted target” network diagram and “compound-potential target” network diagram

The Cytoscape 3.10.1 software was employed to process and analyze the chemical components, targets, and potential targets of SHG. Additionally, a “drug-compound-predicted target” network for SHG and a “compound-potential target” network for SHG in the treatment of AP were constructed.

### 2.5. Construction of protein–protein interaction network

To construct the protein–protein interaction (PPI) network, the potential targets were input into the STRING database^[15]^ website. The resulting PPI network diagram was generated using Cytoscape 3.10.1 software.

### 2.6. Gene Ontology and Kyoto Encyclopedia of Genes and Genomes enrichment analysis

We utilized Rstudio software to conduct Gene Ontology (GO) and Kyoto Encyclopedia of Genes and Genomes (KEGG) pathway enrichment analysis on potential targets.^[16]^

### 2.7. Molecular docking

Download the structure of the chemical components of SHG in TCMSP, obtain the protein structure from the PDB database, use AutoDockTools-1.5.7 for molecular docking, and then use pymol 2.4.0 to visualize the results.^[17,18]^

## 3. Results

### 3.1. The collection of components and targets of SHG

In the TCMSP database, Herb, and literature,^[19,20]^ a total of 65 related components were found in SHG. Among these, 5 components were sourced from *yeyanye*, 12 components from *Inula cappa*, 18 components from *Ardisia japonica*, 17 components from *Bombyx Batryticatus*, 9 components from *Schizonepetae Herba*, and 9 components from *Mentha canadensis*. No relevant components were found in *yangnainaiye*. In addition, there is 1 common component shared by *Inula cappa, Schizonepetae Herba*, and *Mentha canadensis*, 1 common component in *Ardisia japonica* and *Schizonepetae Herba*, and 2 common components shared by *Schizonepetae Herba* and *Mentha canadensis*. After removing duplicate targets from the 65 components, a total of 867 related targets were obtained.

### 3.2. Construction of the “drug-compound-predicted target” network

**Figure 2.**
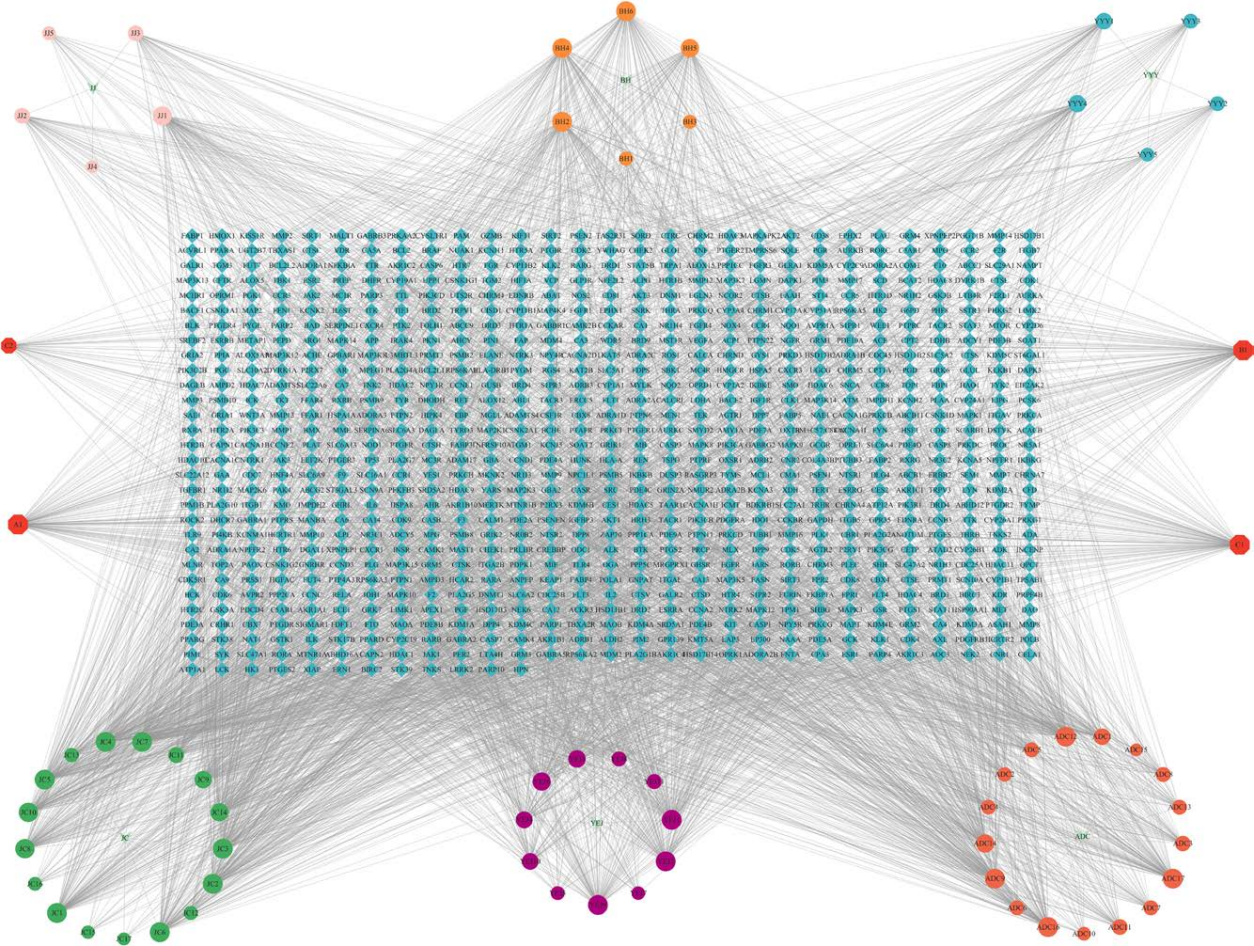
Drug-component-predicted target. In the network graph, diamond shape represents targets, circle shape represents drug component, V-shape represents drug, and octagons on both sides represent the common components.

To visualize the relationship between drugs, components, and predicted targets, Cytoscape 3.10.1 software was employed to construct a “drug-component-predicted target” network (Fig. 2). The network comprises 924 nodes and 3913 edges. The degree value in the network represents the number of edges connected to a node. A higher degree value of a component or a target suggests its potential as a key component or target.

### 3.3. Construction of “ compound-potential-target” network for SHG in the treatment of AP

**Figure 3.**
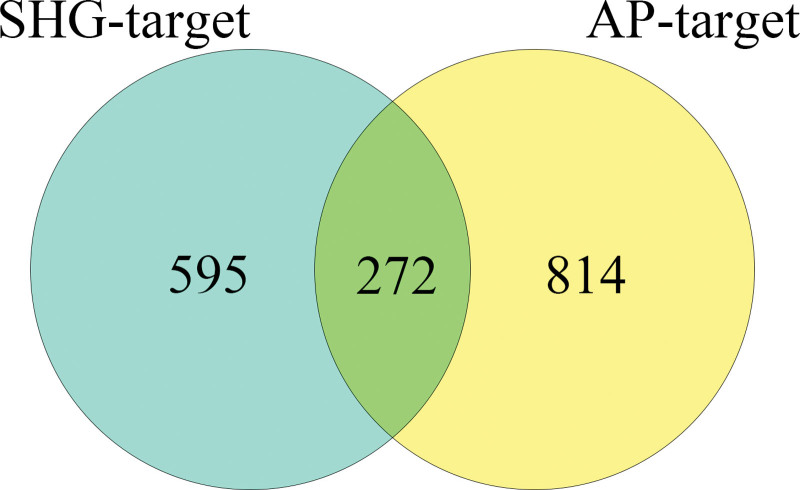
Venn diagram. AP = acute pharyngitis, SHG = Shuangyang houbitong granules.

A total of 2172 AP-related targets were queried in the GeneCards database. Targets with GIFtS values greater than the median were selected as disease targets for AP, resulting in a total of 1086 AP-related disease targets. By intersecting the targets for AP with the predicted targets of the components of SHG, 272 intersection targets were obtained. These intersection targets represent potential target genes for SHG in the treatment of AP (Fig. 3).

**Figure 4.**
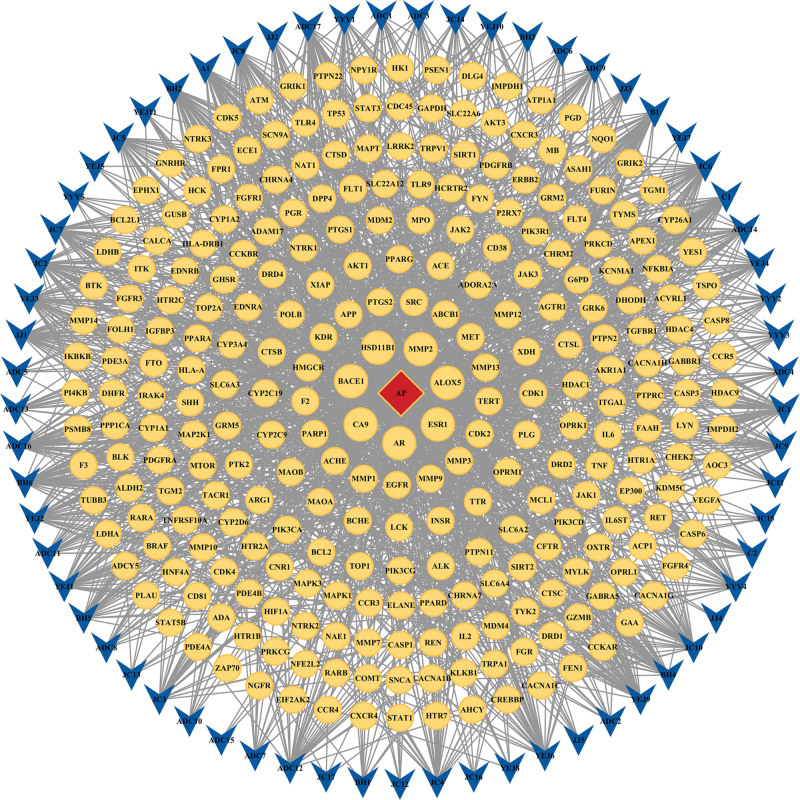
Compound-potential-targets. In this network, the disease is represented by a diamond in the middle, target genes are represented by circles, and components are represented by outer V-shape.

Cytoscape 3.10.1 software was utilized to construct a network called “compound-potential target” network (Fig. 4). This network comprises 338 nodes and 1754 edges. The size of each node corresponds to its degree value. Notably, components such as genkwanin, acacetin, apigenin, and quercetin exhibit higher degree values, suggesting that they could be potential key components. Similarly, target genes like ALOX5, BACE1, ESR1, MMP2, HSD11B1, AR, and CA9 also have greater degree values, indicating their significance in the network.

### 3.4. Construction of PPI network for intersection targets

**Figure 5.**
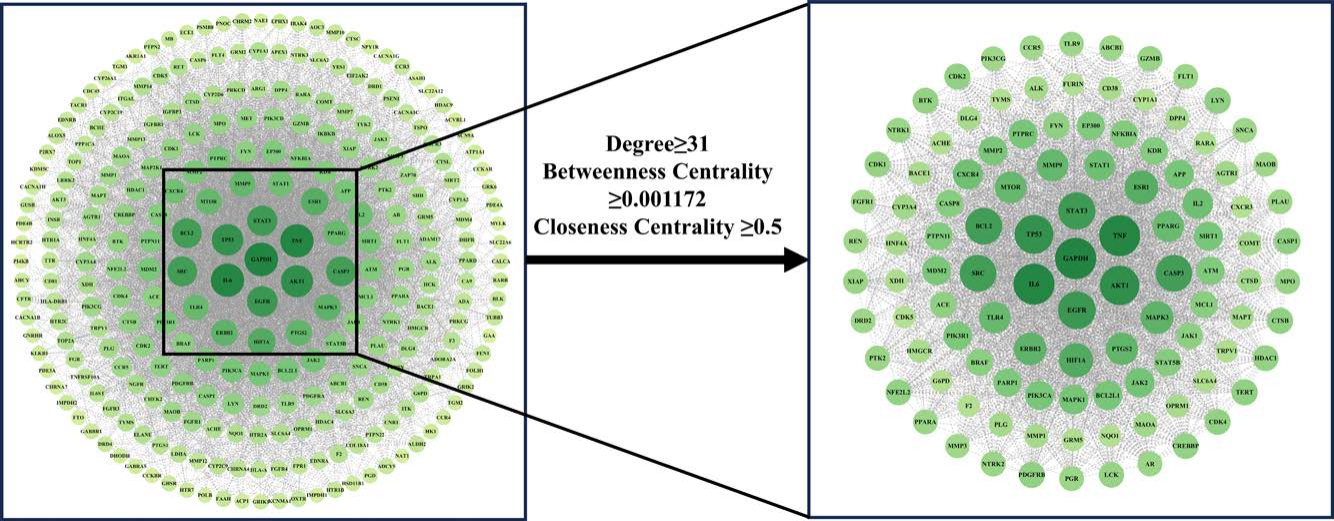
PPI diagram. BC = betweenness centrality, CC = closeness centrality, PPI = protein–protein interaction.

The protein interaction data was imported into Cytoscape 3.10.1 software to construct a PPI network. The size and color of the nodes in the figure were set to be proportional to the Degree value. After performing a topological analysis, targets in the PPI network with degree values, betweenness centrality (BC), and closeness centrality greater than or equal to the median were screened (Fig. 5). Notably, glyceraldehyde 3-phosphate dehydrogenase (GAPDH), tumor necrosis factor (TNF), interleukin 6 (IL6), serine/threonine protein kinase (AKT1), tumor protein 53 (TP53), epidermal growth factor receptor (EGFR), and activator of transcription 3 (STAT3) nodes exhibit higher Degree values and are considered key targets.

### 3.5. KEGG and GO enrichment analysis

**Figure 6.**
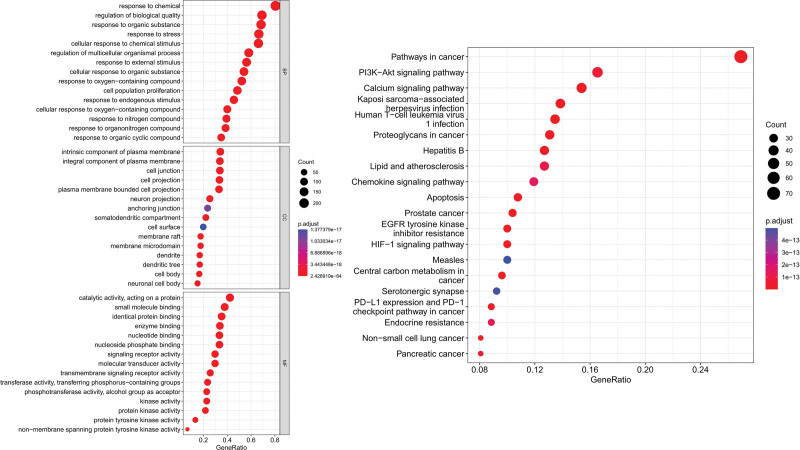
KEGG and GO enrichment analysis. EGFR = epidermal growth factor receptor, GO = Gene Ontology, KEGG = Kyoto Encyclopedia of Genes and Genomes.

A total of 272 potential targets were analyzed for GO and KEGG pathway enrichment (Fig. 6). The GO analysis yielded 3771 items, comprising 3288 biological processes, 184 cellular components (CC), and 299 molecular functions. Additionally, KEGG analysis obtained 182 items.

GO pathway enrichment analysis revealed that SHG participates in biological processes involving response to organic substances, cellular response to chemical stimulus, regulation of multicellular biological processes, cell population proliferation, and cellular response to oxygen-containing compounds. The participating cellular components involve cell junctions, cell projections, neuron projections, anchoring junctions, and membrane rafts, among others. The molecular functions involved include small molecule binding, enzyme binding, nucleotide binding, nucleoside phosphate binding, kinase activity, and protein kinase activity.

**Figure 7.**
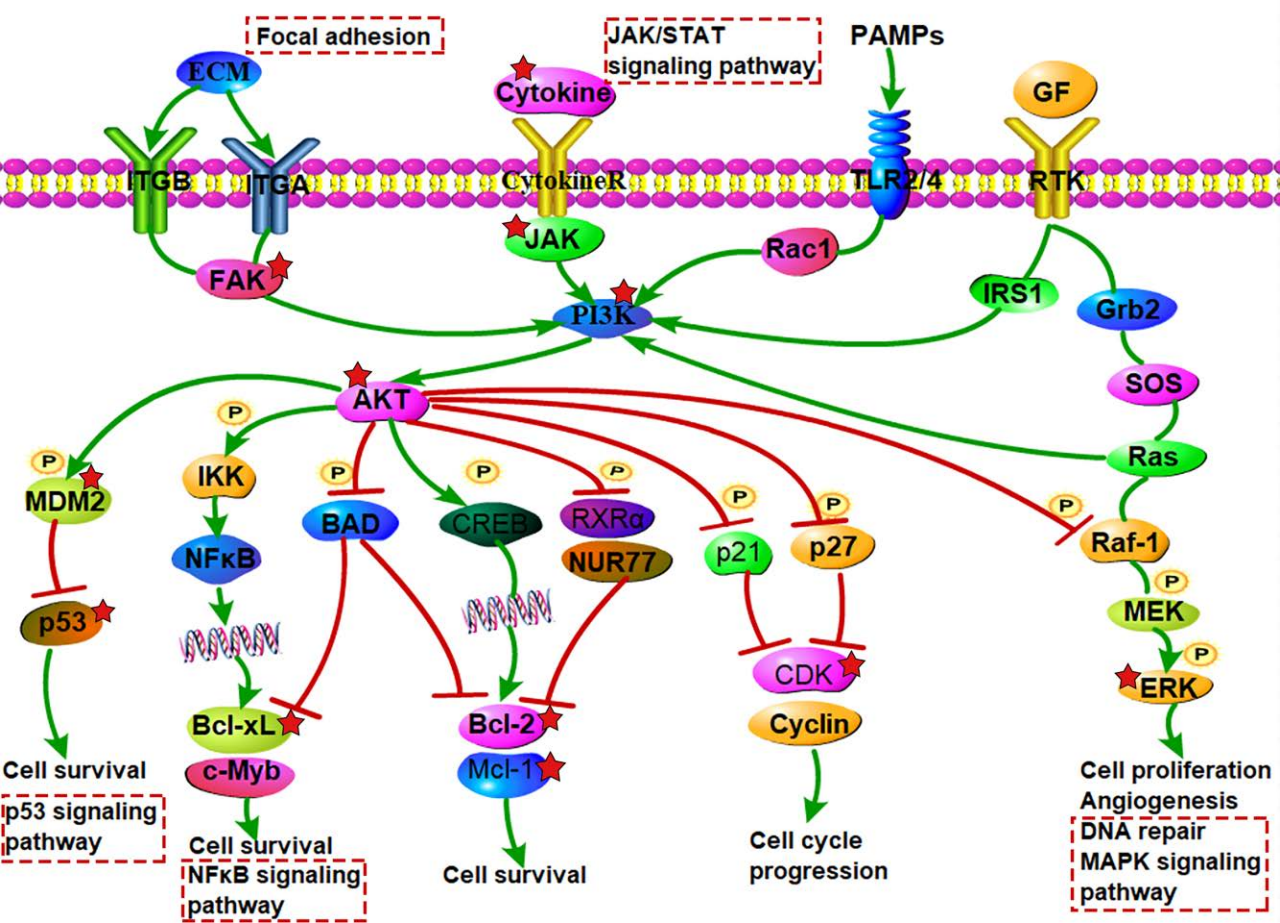
Core pathway of AP intervened by SHG (PI3K-Akt signaling pathway). AP = acute pharyngitis, SHG = Shuangyang houbitong granules.

KEGG pathway enrichment analysis indicated that the PI3K-Akt signaling pathway, Calcium signaling pathway, Kaposi sarcoma-associated herpesvirus infection, Lipid and atherosclerosis, Apoptosis, and HIF-1 signaling pathway may be critical pathways for the treatment of AP by SHG. Among them, the PI3K-Akt signaling pathway ranks highest. Therefore, its mechanism of action is depicted in Figure 7.

### 3.6. Construction of “compound-key target-core pathway” and “core-compound-core key target-core pathway” network for SHG in the treatment of AP

**Figure 8.**
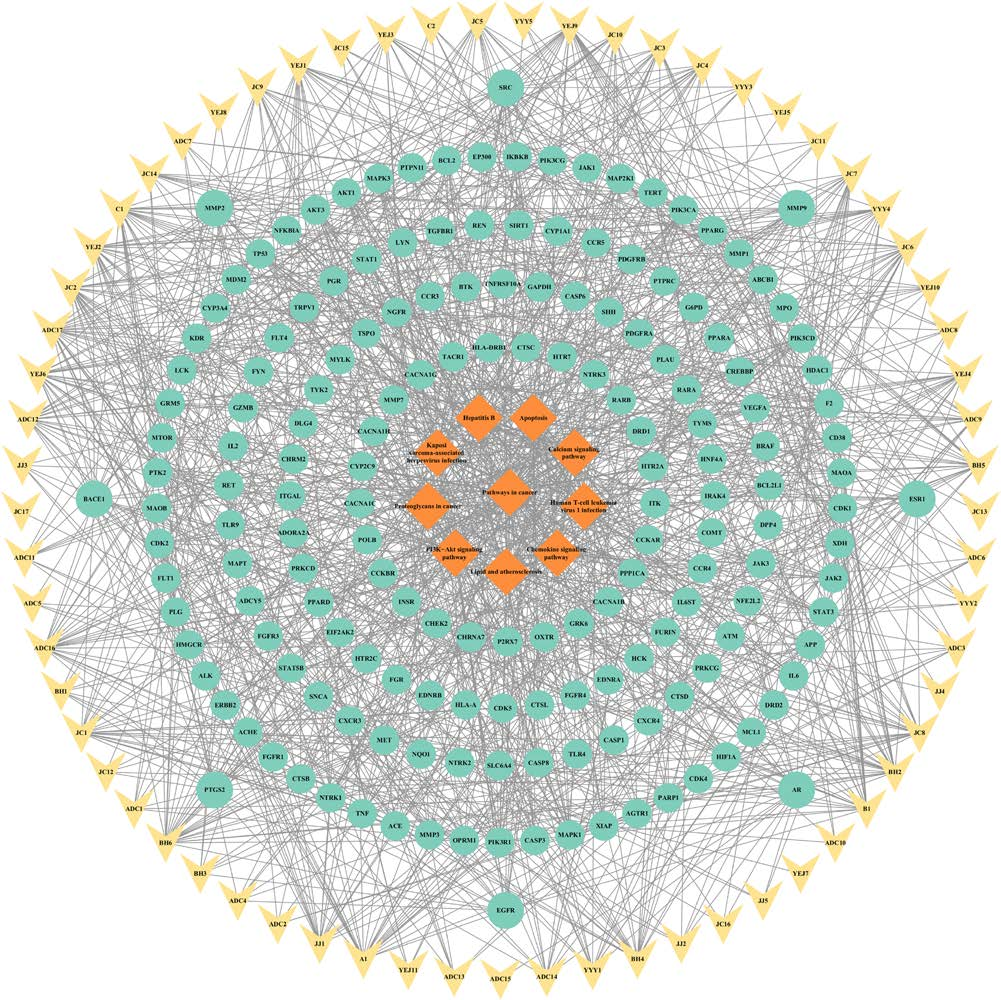
Compound-key target-core pathway. In the network, the diamond symbolizes the core pathway, circle represents the key target, and V-shape represents the drug component.

**Figure 9.**
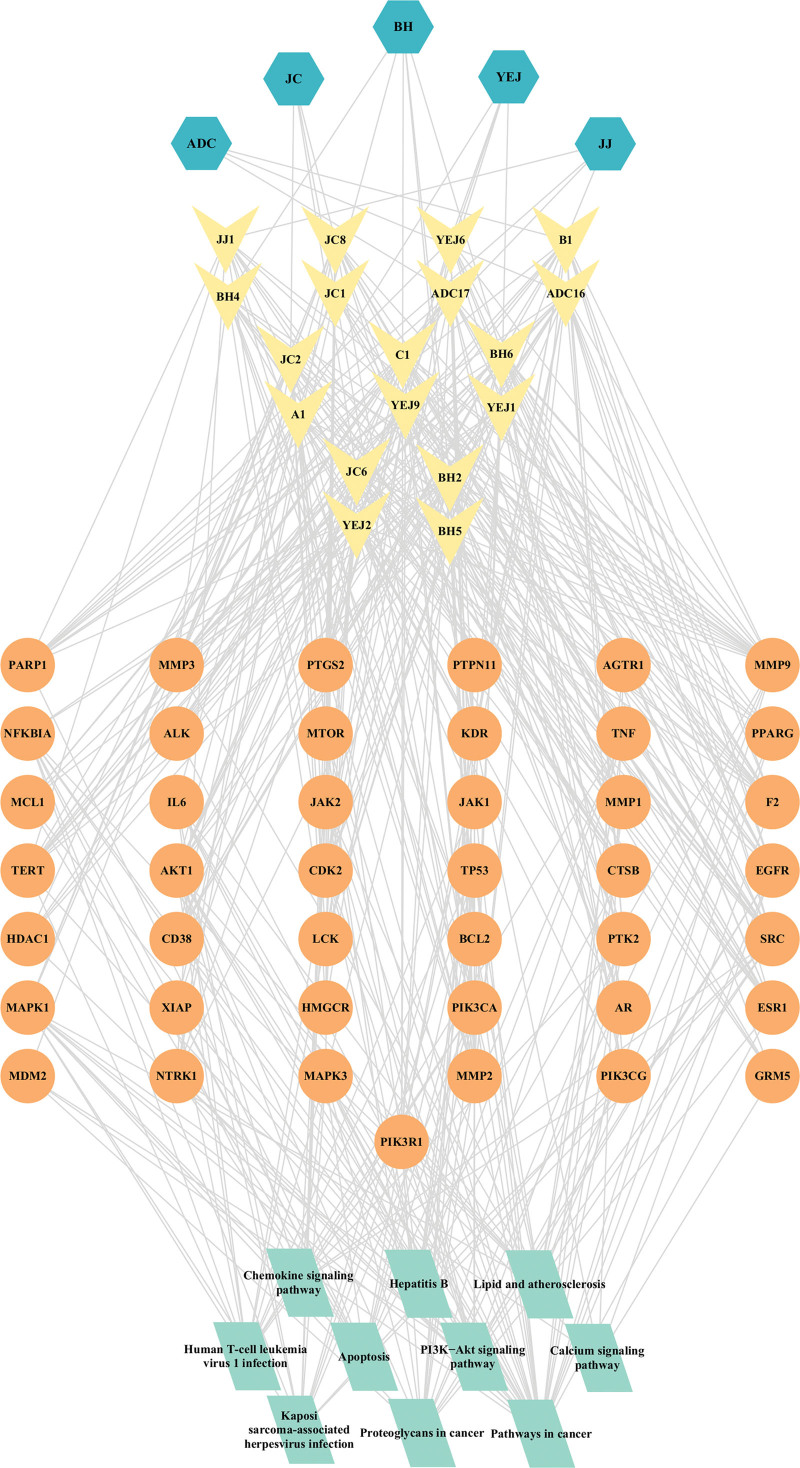
Core-compound-core key target-core pathway. In this network, core pathway is represented by a diamond, target genes are represented by circles, components are represented by outer V-shape and herb are represented by Hexagon.

A “compound-key target-core pathway” network was constructed for the treatment of AP using the top 10 pathways from the KEGG enrichment analysis results (Fig. 8). The network comprises 221 nodes and 928 edges. To identify core compounds, those with BC, closeness centrality, and degree values all greater than the median, and a degree value greater than or equal to 20, are considered. Additionally, targets are selected as core components if their degree values, BC, and closeness centrality are all greater than the median, and their degree value is greater than or equal to 10. Finally, the “core-compound-core key target-core pathway” network can be visualized based on these core compounds and targets (Fig. 9). The network consists of 76 nodes and 446 edges.

### 3.7. Molecular docking

**Figure 10.**
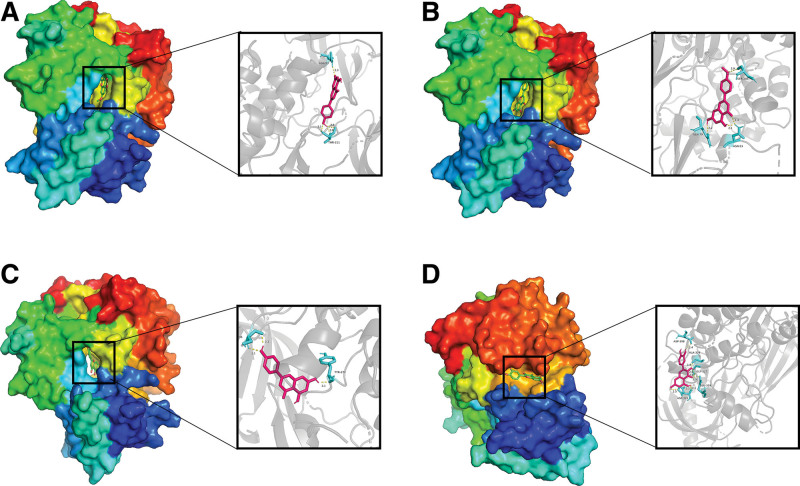
Visualization of molecular docking results. (A) AKT1-Genkwanin, (B) AKT1-Acacetin, (C) AKT1-Apigenin, and (D) AKT1-Qercetin.

In this study, proteins with the highest degree values in the PPI network diagram were selected for molecular docking, namely GAPDH, IL6, TNF, AKT1, TP53, and EGFR. The small molecules chosen were Genkwanin, acacetin, apigenin, and quercetin, as they rank among the top four in terms of degree values in the “compound-potential target” network. Molecular docking was performed using software, and the binding energies are shown in Table 1. The results were evaluated based on the binding energy, where a lower value indicates a better molecular docking outcome. Among them, AKT1 and Genkwanin exhibited the lowest binding energy, measured at −7.59. Four groups with superior combination effects were selected for display, as shown in Figure 10.

**Table 1:** Molecular docking results.

No.	Proteins	Test compounds	Affinity/(kcal/mol)
1	EGFR	Genkwanin	−5.51
2	EGFR	Acacetin	−5.58
3	EGFR	Apigenin	−5.02
4	EGFR	Quercetin	−6.13
5	IL6	Genkwanin	−5.64
6	IL6	Acacetin	−5.7
7	IL6	Apigenin	−5.79
8	IL6	Quercetin	−5.04
9	TNF	Genkwanin	−5.74
10	TNF	Acacetin	−5.93
11	TNF	Apigenin	−5.6
12	TNF	Quercetin	−4.92
13	AKT1	Genkwanin	−7.59
14	AKT1	Acacetin	−7.03
15	AKT1	Apigenin	−6.52
16	AKT1	Quercetin	−5.86
17	TP53	Genkwanin	−5.9
18	TP53	Acacetin	−5.16
19	TP53	Apigenin	−5.96
20	TP53	Quercetin	−4.93
21	GAPDH	Genkwanin	−5.14
22	GAPDH	Acacetin	−5.76
23	GAPDH	Apigenin	−5.6
24	GAPDH	Quercetin	−5.78

AKT1 = serine/threonine protein kinase, EGFR = epidermal growth factor receptor, GAPDH = glyceraldehyde 3-phosphate dehydrogenase, IL6 = interleukin 6, TNF = tumor necrosis factor, TP53 = tumor protein 53.

## 4. Discussion

AP is characterized by an acute inflammation of the pharyngeal mucosa and submucosal tissue, often involving the lymphatic tissue of the pharynx. Clinical manifestations include throat swelling, pain, and difficulty swallowing.^[21,22]^ SHG has been found to have beneficial effects in clearing heat, detoxifying, soothing the throat, and relieving pain.

This study aims to explore the mechanism of action of SHG in treating AP by utilizing network pharmacology combined with molecular docking technology. Through database and literature methods, a total of 65 drug-related components and 867 related targets of SHG were identified. By constructing a “drug-component-potential target” network, genkwanin, acacetin, apigenin, and quercetin were identified as having higher Degree values, indicating their significant role in the treatment of AP with SHG.^[23,24]^

Genkwanin, a methoxyflavone, has been found to possess potent anti-inflammatory effects by inhibiting inflammatory mediators and pathways.^[25]^ Studies have demonstrated that genkwanin can exhibit anti-inflammatory activity by activating B-cell nuclear factor kappa light chain enhancer (NF-κB), nuclear factor erythroid 2-related factor, and transcriptional signal transducer and activator.^[26]^ Additionally, studies have shown that genkwanin can reduce the levels of pro-inflammatory factors TNF-α and IL-6, thereby exhibiting anti-inflammatory effects.^[27]^ Acacetin, a natural brass compound, exhibits various activities including anti-inflammatory, antibacterial, antioxidant, and antiviral properties.^[28]^ Research has revealed that acacetin can inhibit the activity of the SrtA gene in Staphylococcus aureus, suppress the expression of Kv1.3 protein and the influx of Ca^2+,[29]^ and significantly reduce IL-6, IL-8, intercellular adhesion molecule-1, and eosinophils. Moreover, it has been observed that acacetin significantly inhibits the ability of eosinophils to attach to inflammatory BEAS-2B cells. Acacetin also demonstrates a certain effect in inhibiting the secretion of NO and TNF-α.^[30]^ Apigenin, a polyhydroxyflavone, possesses various activities such as anti-inflammatory, antiviral, and antioxidant effects.^[31]^ Studies have indicated that apigenin down-regulates monocyte inflammatory protein-1α, intracellular adhesion molecules, and monocyte chemotaxis through extracellular signal-regulated kinase and mitogen-activated protein kinase. This leads to the suppression of inflammatory cytokine expression in the body, thereby achieving anti-inflammatory purposes. Additionally, apigenin has been reported to inhibit mTOR activation by downregulating the PI3K/AKT/MTOR pathway.^[32]^ Apigenin can also prevent the expression of STAT3 and STAT5 genes and enhance the activities of STAT1, STAT2, and LMWPTP, thereby inhibiting cell proliferation.^[33]^ Quercetin, a polyhydroxyflavone, exerts anti-inflammatory effects by inhibiting inflammatory factors and enzymes.^[34]^ It mainly reduces pro-inflammatory cytokines IL-1β and IL-6 while increasing anti-inflammatory cytokines IL-4, IL-10, and Transforming growth factor-β1. Additionally, it inhibits NF-κB and mitogen-activated protein kinase signaling pathways to exert anti-inflammatory effects.^[35]^

Through PPI network and molecular docking verification, it was concluded that GAPDH, IL6, TNF, AKT1, TP53, and EGFR may be the key targets of SHG in treating AP. GAPDH, a glycolytic enzyme, plays a crucial role in sugar metabolism in the human body. It is expressed in nearly all cells and is involved in various non-glycolytic processes, including apoptosis, tumor formation, DNA repair, and cell membrane fusion.^[36]^ IL-6, a pleiotropic cytokine, plays a crucial role in autoimmune mechanisms, acute inflammation, and the progression to chronic inflammatory states.^[37]^ It also plays a significant role in B cells, inducing plasma cell differentiation and antibody production.^[38]^ Studies have shown that pharyngitis can cause a significant increase in IL-6 levels, and reducing IL-6 levels can alleviate pharyngitis.^[39]^ AKT1, a protein kinase, is involved in various cell signaling mechanisms related to cell metabolism, growth, division, apoptosis inhibition, and angiogenesis.^[40]^ Research has demonstrated a close association between AKT1 and inflammatory responses.^[41]^ TNF plays a crucial role in mediating and regulating immune responses in both healthy individuals and those with diseases. It controls various aspects such as the development of the immune system, signaling pathways for cell survival, proliferation, and metabolic processes. Research has indicated that patients with AP exhibit increased levels of TNF in their serum.^[42]^ Moreover, it has been found that reducing TNF levels can effectively alleviate pharyngitis symptoms. EGFR is a growth factor receptor that induces cell differentiation and proliferation upon activation through the binding of one of its ligands.^[43]^ TP53 is a crucial molecule in the realm of cell cycle research. Recent studies conducted on in vitro cells have demonstrated that inhibiting TP53 can enhance the proliferation of human nucleus pulposus cells.^[44]^

The results of the GO pathway enrichment analysis indicate that SHG can regulate cell population proliferation, cell response to chemical stimulation, and other processes by modulating nucleotide binding, nucleoside phosphate binding, and enzyme binding to treat AP. The KEGG pathway enrichment analysis reveals that the targets are mainly enriched in various pathways such as the PI3K-Akt signaling pathway, calcium signaling pathway, Kaposi’s sarcoma-associated herpes virus infection, and HIF-1 signaling pathway. The PI3K-Akt pathway plays a crucial role in cell senescence, apoptosis, cell proliferation, migration, and inflammatory responses.^[45]^ Studies have shown that regulating the PI3K-Akt pathway can inhibit inflammatory factors and reduce inflammatory reactions,^[46]^ suggesting that SHG may effectively treat AP by modulating this pathway.

As a novel approach to studying drug actions and mechanisms, network pharmacology has many unique advantages. However, the limitations of network pharmacology should not be overlooked. For example, the accuracy and completeness of databases need to be improved, data mining capacity is limited, and the results of network pharmacology require experimental validation. Despite these limitations, network pharmacology, as an emerging technology for studying drug actions, provides valuable information for drug discovery and target identification. With advancements in database quality and computational models and algorithms, it is expected that network pharmacology will play an increasingly significant role in future drug research and discovery.

## 5. Conclusion

In conclusion, this study utilized network pharmacology and molecular docking technology to identify the core components of SHG in treating AP as genkwanin, acetin, apigenin, and quercetin, with GAPDH, IL6, TNF, AKT1, TP53, and EGFR being the core targets. Additionally, SHG was found to regulate multiple pathways including the PI3K-Akt signaling pathway, calcium signaling pathway, and HIF-1 signaling pathway. Furthermore, SHG was observed to modulate processes such as nucleotide binding, nucleoside phosphate binding, and enzyme binding, which are involved in regulating cell population proliferation and cell response to chemical stimulation. These findings highlight the potential of SHG in the treatment of AP. The findings provide a theoretical basis and offer insights for further clinical research.

## Acknowledgements

We are very grateful for the contributions of TCMSP database, HERB database, China National Knowledge Infrastructure, SwissTargetPrediction database, STRING database, Pubchem database and Genecards database that provide information on research, as well as all colleagues involved in the study.

## Author contributions

**Conceptualization:** Jiying Zhou, Jiarui Wu.

**Data curation:** Jiying Zhou, Chuanqi Qiao, Haojia Wang.

**Funding acquisition:** Jiying Zhou, Jiarui Wu.

**Supervision:** Yifei Gao, Jiaqi Li.

**Visualization:** Jiying Zhou, Chuanqi Qiao, Keyan Chai, Tong Zhao.

**Writing – original draft:** Jiying Zhou.

**Writing – review & editing:** Haojia Wang, Siyun Yang, Jiarui Wu.
